# Gold nanoparticle clusters for the investigation of therapeutic efficiency against prostate cancer under near-infrared irradiation

**DOI:** 10.1186/s40580-019-0216-z

**Published:** 2020-02-17

**Authors:** Jeonghun Kim, Sang Hun Chun, Lunjakorn Amornkitbamrung, Chanyoung Song, Ji Soo Yuk, So Yeon Ahn, Byung Woo Kim, Yong Taik Lim, Byung-Keun Oh, Soong Ho Um

**Affiliations:** 1 School of Chemical Engineering, Sungkyunkwan University, Suwon, Gyeonggi-do 16419 South Korea; 2 SKKU Advanced Institute of Nanotechnology (SAINT), Sungkyunkwan University, Suwon, Gyeonggi-do 16419 South Korea; 3 Polymer Engineering Laboratory, Department of Chemical Engineering, Faculty of Engineering, Chulalongkorn University, 254 Phayathai Road, Pathumwan, Bangkok, 10330 Thailand; 4 Biomedical Institute for Convergence at SKKU (BICS), Sungkyunkwan University, Suwon, Gyeonggi-do 16419 South Korea; 5 Department of Chemical and Biomolecular Engineering, Sogang University, Seoul, 04107 South Korea

**Keywords:** Gold nanoparticle cluster, Photothermal therapy, Prostate cancer treatment

## Abstract

Gold particles have been widely used in the treatment of prostate cancer due to their unique optical properties, such as their light-heat conversion in response to near-infrared radiation. Due to well-defined synthesis mechanisms and simple manufacturing methods, gold particles have been fabricated in various sizes and shapes. However, the low photothermal transduction efficiency in their present form is a major obstacle to practical and therapeutic uses of these particles. In the current work, we present a silica-coated gold nanoparticle cluster to address the therapeutic limit of single gold nanoparticles (AuNPs) and use its photothermal effect for treatment against PC-3, a typical prostate cancer. Due to its specific nanostructure, this gold nanocluster showed three times higher photothermal transduction efficiency than free single AuNPs. Moreover, while free single particles easily clump and lose optical properties, this silica-coated cluster form remained stable for a longer time in a given medium. In photothermal tests under near-infrared radiation, the excellent therapeutic efficacy of gold nanoclusters, referred to as AuNC@SiO_2_, was observed in a preclinical sample. Only the samples with both injected nanoclusters followed by photothermal treatment showed completely degraded tumors after 15 days. Due to the unique intrinsic biocompatibility and higher therapeutic effect of these silica-coated gold nanoclusters, they may contribute to enhancement of therapeutic efficacy against prostate cancer.

## Introduction

Prostate cancer is one of the most globally prevalent types of cancer, even though it only affects the male population. It is a common cancer in the United States and its incidence is increasing every year [1, 2]. Although there are various treatments, radiation therapy is most commonly used. However, it is often fatal to elderly patients and prostatectomy can cause serious problems such as incontinence and erectile dysfunction [3, 4]. Androgen deprivation therapy presents the patient with a risk of developing prostate cancer and is not used [3].

Recently, new therapeutic techniques have been developed using combinations of various organic and inorganic materials. Efforts are being made to overcome the disadvantages (e.g., the side effects of drugs in a body) of existing single drug and surgical treatments [4–12]. Among these, some photothermal agents have attracted much attention. They can be used in treating prostate cancer by converting light into heat energy under laser irradiation resulting in thermal destruction of cancer cells at treatment sites [13–23]. One of the great advantages of photothermal therapy (PTT) is done through the use of near-IR (NIR) radiation. NIR is not absorbed by biological materials or water [24]. This behavior allows efficient removal of tumor cells in deep tissues [25]. It has been developed into a selective and powerful tool without some of the disadvantages of conventional noninvasive therapies such as radiotherapy, chemotherapy and microwave therapy [26, 27]. Currently, photothermal agents made of plasmon nanoparticles have been developed. Some of them have been used in NIR investigations and have become an ideal prostate cancer treatment [28, 29]. In particular, gold particles have been widely used due to their biocompatibility and unique optical characteristics that include surface plasmon resonance (SPR). SPR is a phenomenon in which incident light of a specific wavelength excites free electrons causing resonance of metal particles on the surface their nanoparticles. These features vary depending on size and shape of the metal particles. Altering particle size and shape can be used to express the unique characteristics these metal particles [13, 14]. Gold nanoparticles are often used as a photothermal agents because of their well-defined and simple synthesis methods. However, gold nanoparticles have several disadvantages in practical PTT use. Therefore, photothermal agents are being developed to improve the efficiency of photothermal transfer, while maintaining the unique optical properties of AuNPs. Among the strategies used to modify these properties, clustering due to resonance interference between nanoparticles is being examined [30–33]. They can be used a nucleus of seed particle assembly. Also, the collective effect of AuNPs is achieved through resonance interference can be changed by adjusting their optical properties. This ultimately amplifies the photothermal effect in the NIR region [34–36]. Inspired by these interesting properties, gold nanoparticle clusters (AuNCs) can be applied as an efficient photothermal agents [37–40].

**Scheme 1:**
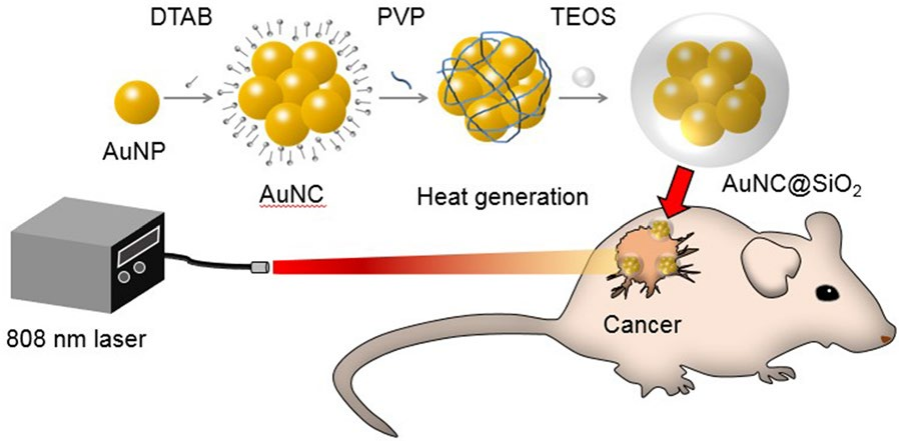
Schematic drawing of gold nanocluster-induced photothermal treatment in vivo. To confirm the therapeutic efficiency, AuNC@SiO_2_ was used to treat prostate tumors using the irradiation of near-infrared laser

A method for building AuNPs in cluster form is using a cationic surfactant and biocompatible polymer [40]. In this approach, AuNCs were fabricated via a simple process while maintaining their strong performance as a photothermal agents. In this study, we investigated a new photothermal agent that can be used in the treatment of in vitro (Scheme 1) and in vivo cancers. AuNC@SiO_2_ with a diameter of 60–70 nm was synthesized as previously reported [40]. The synthesized AuNC@SiO_2_ showed higher photothermal transduction efficiencies of 11.29% to 36.21% over that of single AuNPs. To test the internalization of AuNC@SiO_2_ in a prostate cancer cell line (PC-3), fluorescent dye was incorporated into the silica layer of the AuNC@SiO_2_. Observations confirmed that it remained inside the PC-3 cells. In this study, it was found that cytotoxicity was not observed but the PTT showed a resultant survival of PC-3 cells of 20%. The thermal conversion efficiency of AuNC@SiO_2_ was very good. The tumors completely disappeared within 15 days.

## Experimental methods

### Materials

Tetrachloroauric acid trihydrate (HAuCl_4_·3H_2_O, 99.9%), trisodium citrate dihydrate (99%), dodecyltrimethylammonium bromide (DTAB, 98%), polyvinylpyrrolidone (PVP, Mw ~ 55,000), ethylene glycol (EG, 99.8%), ammonium hydroxide (28–30% NH_3_ basis), tetraethyl orthosilicate (TEOS, 99%), dimethyl sulfoxide (DMSO, 99.9%), fluorescein isothiocyanate isomer (FITC, 90%), and (3-aminopropyl) triethoxysilane (APTES, 99%) were purchased from Sigma Aldrich. Fetal Bovine Serum (FBS), Trypsin–EDTA (0.25% solution), a penicillin streptomycin solution, RPMI 1640 media, and phosphate buffered saline (PBS) were purchased from Gibco. 3-(4,5-dimethylthiazol-2-yl)-2,5-diphenyltetrazolium bromide (MTT) was purchased from Thermofisher. Deionized water (18.2 MΩ/cm) and ethanol (EtOH, 99%) were used as solvents. All reagents were used without further purification.

### Synthesis of AuNC@SiO_2_

AuNC@SiO_2_ was prepared as previously described [40]. Briefly, AuNPs were fabricated via a standard sodium citrate reduction method. 100 mL of HAuCl_4_ was stirred at 100 °C and 10 mL of aqueous tri-sodium citrate dehydrate (38.8 mM) was added to the solution. This mixture was continuously stirred for 15 min and then cooled to room temperature. AuNCs were synthesized using AuNPs as a precursor. Typically, 1 mL of a DTAB aqueous solution (20 mg/mL) and 1 mL of an aqueous AuNP (12 nM) solution were mixed. The resulting solution was agitated for few seconds using a vortex mixer and swiftly added into 5 mL of a PVP/EG solution (2 mM). The mixture was stirred at 700 rpm for 30 min. The obtained AuNCs were washed twice after centrifugation at 15,000*g* for 20 min and re-dispersed in 1 mL of ethanol. After that, the clusters were coated with silica using a sol–gel process [41, 42]. 1 mL of AuNC in ethanol was added into 9 mL of ethanol with stirring at 700 rpm. Then, 1.5 mL of deionized water and 0.5 mL of an ammonium hydroxide solution were added dropwise into the solution. Finally, 10 μL of TEOS was swiftly added and the mixture was continuously stirred at room temperature for 30 min. The resulting AuNC@SiO_2_ was washed five times after centrifugation using ethanol and finally re-dispersed in 1 mL of deionized water.

### Synthesis of FITC-AuNC@SiO_2_

FITC was used to confirm intracellular infusion of AuNC@SiO_2_. 19.5 mg of FITC, 11.7 μL of APTES and 50 mL of ethanol were stirred in a dark environment at 42 °C for 24 h. Then, this solution prepared was used to wash the AuNC@SiO_2_ instead of ethanol, sandwiching FITC-APTES between silica particles. The synthesized complex was washed five times with water and the supernatant containing unreacted FITC-APTES was collected after centrifugation. After measuring the fluorescence of the supernatant, it was confirmed that unreacted FITC-APTES was completely removed by washing four times (Additional file 1: Figure S5C).

### Characterization

The characteristics of the synthesized AuNCs were investigated using UV–vis spectroscopy and dynamic light scattering (DLS). A SpectraMax M5 microplate reader (Molecular Devices, Sunnyvale, CA, USA) was used for spectroscopic analysis employing disposable cuvettes as a sample holders. The particle size was measured using DLS (Malvern, Zetasizer Nano ZS90). The morphology of AuNCs was visualized using transmission electron microscopy (TEM, JEOL JEM-3010) operating at 120 kV. Samples (10 μL) were dropped on to 200 mesh carbon-coated copper grids and dried at room temperature.

### *In*-*vitro* analysis

A prostate cancer cell line (PC-3) was cultured in a complete medium (RPMI media mixture containing 10% of FBS, 1% of penicillin, and 1% of streptomycin) at 37 °C under a 5% CO_2_ humidified air environment [43]. 100 μL of fresh RPMI media solution was added into a 96-well plate containing 2 × 10^4^ cells in each well and it was incubated overnight. Each nanoparticle dispersion was incubated for 1–3 h. After that, the plate was removed from the incubator and it was washed with PBS three times to remove residues. An additional step was done to detach the cells from the well plate so that they could be characterized in further analysis (FACS, MACS Quant VYB). In this step, trypsin–EDTA was used, followed washing three times with PBS and centrifugation. The samples were re-dispersed in 200 μL of a 4% paraformaldehyde PBS-based solution for fixation. After 10 min, the samples were washed twice with PBS and stored in PBS. Fluorescence of cells was measured using FACS and fluorescence microscopy (Axiovert 200 M, ZEISS, Germany). PC-3 cells were grown exposed to each of the nanomaterials in the current study for 24 h to determine the cytotoxicity of the nanomaterials. After laser irradiation, viability tests were performed. Here, the medium containing gold nanomaterials was removed and the cells were washed several times. The MTT assay was done to investigate cell viability. After incubation of cells with 10 μL of MTT for an hour, MTT formazan was formed and dissolved with 100 μL of DMSO. The solution was centrifuged three times and the cells re-suspended in DMSO to quantify the MTT formazan produced by living cells. Absorbance of the supernatant at 540 nm was measured. This was done to remove the remaining Au particles so they could not interfere with measurements of MTT formazan production.

### In-vivo analysis

BALB/c-nude mice were employed to investigate the in vivo photothermal effect of AuNC@SiO_2_. Twelve female mice, aged 10–16 weeks, were used. The initial weight of these mice was around 25–30 g. They were subcutaneously injected in the flank with 10^7^ of PC-3 cells that had been re-suspended in 100 μL of PBS. Tumors were allowed to grow for around 2 weeks before starting the experiment. The initial volume of tumors was about 100 mm^3^. At this point, mice were treated with sample injections followed by laser irradiation. The mice were anesthetized using 2,2,2-tribromoethanol. This animal study was reviewed and approved by the Institutional Animal Care and Use Committee (IACUC) of Sungkyunkwan University School of Medicine, which is accredited by the Association for Assessment and Accreditation of Laboratory Animal Care International (AAALAC International) and abides by the Institute of Laboratory Animal Resources (ILAR) guide.

### Investigation of laser treatment

The photothermal effect of irradiated AuNC@SiO_2_ was investigated using an 808 nm wavelength NIR laser. Each sample (10 μg) was loaded into a well of a 96-well plate for the in vitro experiment. In the in vivo experiment, a 30 μg sample of AuNC@SiO_2_ was subcutaneously injected in the flanks of mice. After 3 h, the mice were irradiated at the tumor site for 3 min using a laser with a power of 2.5 W/cm^2^ (measured with a power meter). The laser beam was generated by a fiber coupled 808 nm laser diode. The spot size was set to about 25 mm^2^ to irradiate the tumor area.

## Results and discussion

**Fig. 1:**
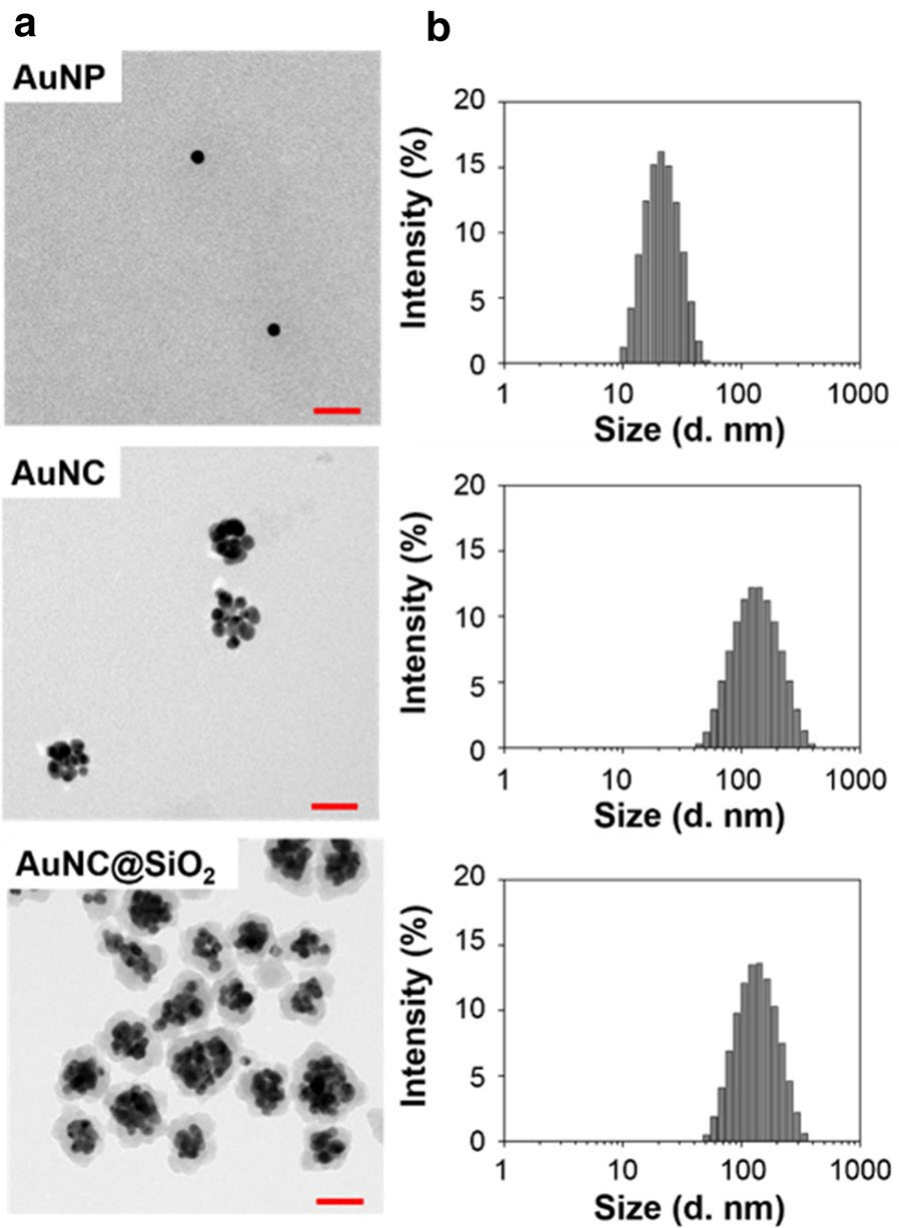
Size and shape confirmation of each Au nanoparticle. **a** TEM images of AuNP, AuNC and AuNC@SiO_2_. The size of scale bars is 50 nm. **b** Particle size distribution histograms of AuNP, AuNC, and AuNC@SiO_2_, from the upper to lower right. Each DLS data point is an average of 15 scans

Prior to testing the therapeutic efficacy of the synthesized gold nanoparticle clusters, AuNC@SiO_2_ was prepared as described previously [40] and then confirmed using several other methods. Changes in the morphology during conversion from AuNP to AuNC and AuNC@SiO_2_ were confirmed through TEM observations as shown in Fig. 1a. The average AuNP size was 13.44 ± 1.32 nm and that of AuNC was 52.01 ± 3.73 nm. After coating the AuNPs with silica, their diameter increased to 66.47 ± 5.59 nm, due to the presence of silica layers on their surfaces. The hydrodynamic diameter distribution of the particles was measured using DLS and the results are shown in Fig. 1b. The DLS data for the AuNP, AuNC, and AuNC@SiO_2_ particles showed average sizes of 19.3, 107.7, and 116.8 nm, respectively. DLS showed larger sized particles compared to TEM, due to a hydrated layer around the particles. The lower PDI (poly-dispersive index) value of each sample (0.189 ± 0.0026 for AuNP, 0.296 ± 0.0100 for AuNC, and 0.238 ± 0.0132 for AuNC@SiO_2_) indicated their uniformity. In addition to size confirmation, the surface charges of gold nanoparticle clusters were measured. As shown in Additional file 1: Figure S1, the zeta potential of the AuNPs (− 25.1 ± 4.59 mV) was strongly influenced by citrate groups, resulting in a strong negative charge. Once in AuNC form, the value was closer to neutral at − 8.54 ± 0.62 mV due to its surface stabilization by PVP. After the AuNCs were silica-coated, a strong negative charge (− 24 ± 1 mV) was measured due to the presence of silanol groups on their surfaces. The optical characteristics also changed depending on the particle species. The absorption spectrum in the UV–vis region was measured for AuNP, AuNC and AuNC@SiO_2_ (Additional file 1: Figure S2). The results showed a shift of absorption peaks from shorter to longer wavelengths as they were transformed from AuNPs to AuNCs and further to AuNC@SiO_2_. AuNPs had a strong absorption peak at 520 nm. Alternatively, both the AuNCs and AuNC@SiO_2_ had an absorption peak at 540 nm with peak broadening. This can be attributed to aggregation of AuNPs that was induced by plasma mode coupling. In the case of AuNC@SiO_2_, the peak was much broader than that of AuNCs due an increased local refractive index around the clusters caused by the silica layer. Additionally, the absorption in the NIR region (800–900 nm) of AuNCs was 2.3 times higher than that of AuNPs, while that of AuNC@SiO_2_ was 5.2 times higher than free AuNPs. This indicates that when NIR lasers irradiate AuNCs and AuNC@SiO_2_, a larger amount of light is converted into heat. The η value is the photothermal transduction efficiency. This parameter is often used as an index representing the thermal conversion efficiency of a material by a photothermal effect. The photothermal transduction efficiency of each material was calculated from the experimental results shown in Additional file 1: Figure S3A, B. The energy balance equations (1–10) are given in Additional file 1 [44, 45]. In the measurement of the photothermal transduction efficiencies using an 808 nm NIR laser (Additional file 1: Figure S3A, B), η of the AuNPs was 11.29%. However, when it was aggregated by PBS, the efficiency increased to 34.58%. Also, it was confirmed that the photothermal transduction efficiency of AuNCs was 30% and that of AuNC@SiO_2_ was 36.21%. At the moment of aggregation of AuNPs into clusters, more efficient photothermal conversion was confirmed for the same amount of material and energy source.

**Fig. 2:**
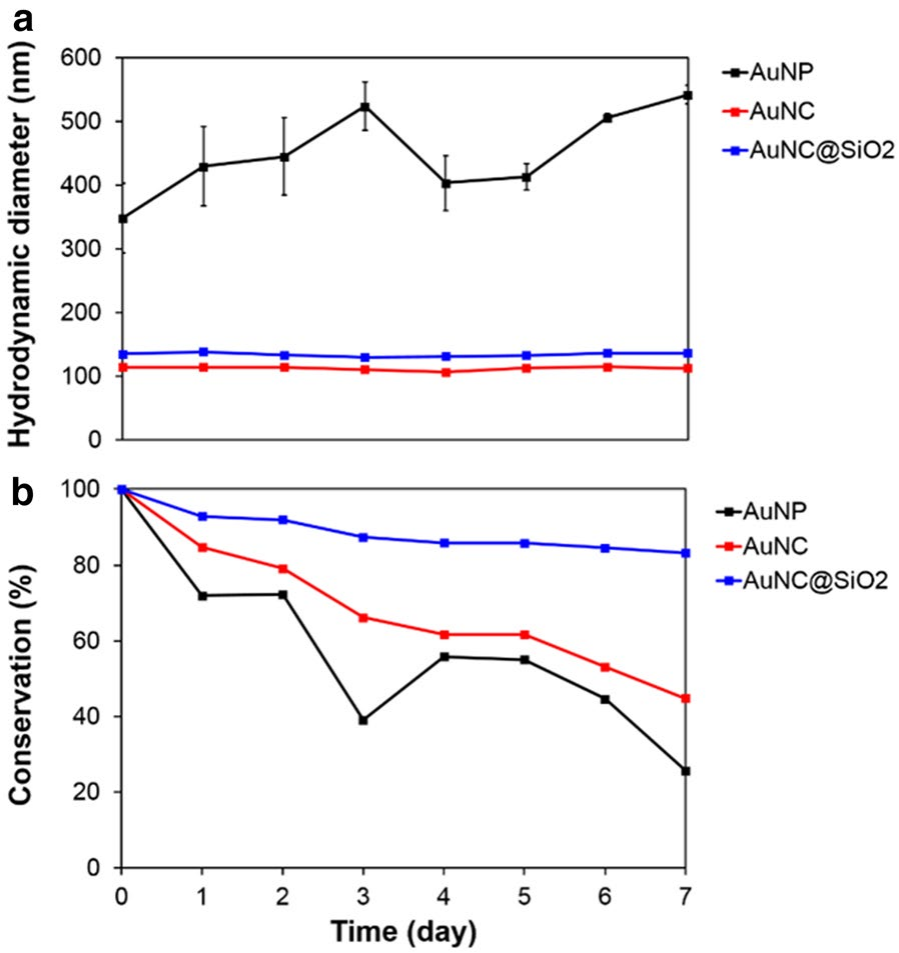
Stability experiment of each Au nanoparticle type in PBS solution. **a** Size variation of each Au nanoparticle with time and **b** absorption change of OD values at 800 nm obtained using a microplate reader. Each sample was scanned 6 times. DLS experimental data were obtained through triplicate measurements

In Fig. 2, the particle stability in PBS was investigated by observing changes in size and optical properties over time. PBS is the most representative balanced buffer that can be used in place of a biological fluid. In PBS, the size of AuNPs dramatically increased to around 400 nm and fluctuated over time. Also, the optical properties of AuNPs were greatly changed. The absorbance peak at 520 nm disappeared when AuNP were stored in PBS. This indicated aggregation of AuNP in PBS. It is well established that citrate stabilized AuNPs are negatively charged and they can be destabilized by positively charged ions such as calcium and magnesium that are present in the buffer. Alternatively, AuNCs and AuNC@SiO_2_ showed sustained particle sizes in pure water and even PBS due to the physical stability of the PVP and silica protected surfaces. The optical properties of AuNCs in Additional file 1: Figure S4 indicate that they had a specific absorbance peak, but their stability in PBS decreased over time. AuNC@SiO_2_ was most stable in PBS and its absorbance was almost unchanged over time. As a result, the stability of AuNC@SiO_2_ proved to be excellent. Its silica coating is not reactive in living tissue so there is a low risk of vascular absorption.

**Fig. 3:**
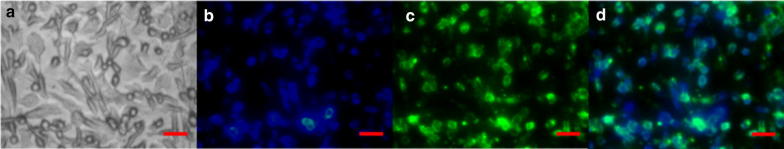
Evaluation of the cell internalization of FITC-AuNC@SiO_2_. PC-3 cells were treated and incubated with 2 μg of FITC-AuNC@SiO_2_ 3 h at 37 °C. The cells were then washed, incubated with DAPI for nuclei-staining, and analyzed using fluorescence microscopy. Bright field image (**a**), blue fluorescence image of PC-3 cells for nuclei-staining DAPI (**b**), green fluorescence image of PC-3 cells for FITC (**c**). **d** is a merged image of **b** and **c**. The size of scale bars is 100 μm

To achieve efficacy in photothermal therapy (PTT), it is essential that the photothermal agent can easily be internalized by the target cells. FITC dye was incorporated into AuNC@SiO_2_ to assess this by visualizing the cellular uptake of the photothermal agents. FITC-AuNC@SiO_2_ was synthesized by simply mixing FITC-APTES and AuNC during the silica coating process (Additional file 1: Figure S5A). The formation of FITC-AuNC@SiO_2_ complexes was confirmed by measuring their absorption spectrum. The UV–vis spectrum of FITC-APTES exhibited an absorption peak at around 490 nm, while AuNCs had an absorption peak at 540 nm (Additional file 1: Figure S5B). After FITC was introduced into the silica layer of the AuNCs, their spectrum showed two wide peaks at around 490 nm and 540 nm, indicating that FITC was attached to the AuNCs. The fluorescence intensity was measured to confirm the formation of FITC-AuNC@SiO_2_ complexes. Additionally, FITC-AuNC@SiO_2_ precipitates had fluorescence signals indicating that FITC dye was successfully incorporated into the silica layer. Cellular internalization of FITC-AuNC@SiO_2_ was then investigated by exposing PC-3 cells, the typical prostate cancer selected as a test model in this study, to FITC-AuNC@SiO_2_. The results shown in Fig. 3 can be used to confirm by fluorescence microscopy that cellular internalization of FITC-AuNC@SiO_2_ was achieved. The cell nuclei were stained with a DAPI dye that exhibits a blue fluorescence (Fig. 3b), and FITC fluorescence of AuNC@SiO_2_ is shown in green in Fig. 3c. In Fig. 3d, the images in Fig. 3b, c are combined, showing an overlap of the blue and green fluorescence. This indicates that FITC-AuNC@SiO_2_ was internalized in the PC-3 cells. FITC-AuNC@SiO_2_ was successfully internalized into PC-3 cancer cells. FACS was used to measure the cell penetration rate of particles and the results are shown in Additional file 1: Figure S6. The mean fluorescence value was 436.3 ± 25.6 after 1 h, which increased to 1062 ± 14.2 after 2 h of culture. It further increased to 1240.3 ± 86.5 after 3 h. As incubation time was increased, larger numbers of particles were introduced into the cells. However, the difference between 2 and 3 h was clearly smaller than the difference from 1 to 2 h. This data further supports that AuNC@SiO_2_ entered well into the cells.

**Fig. 4:**
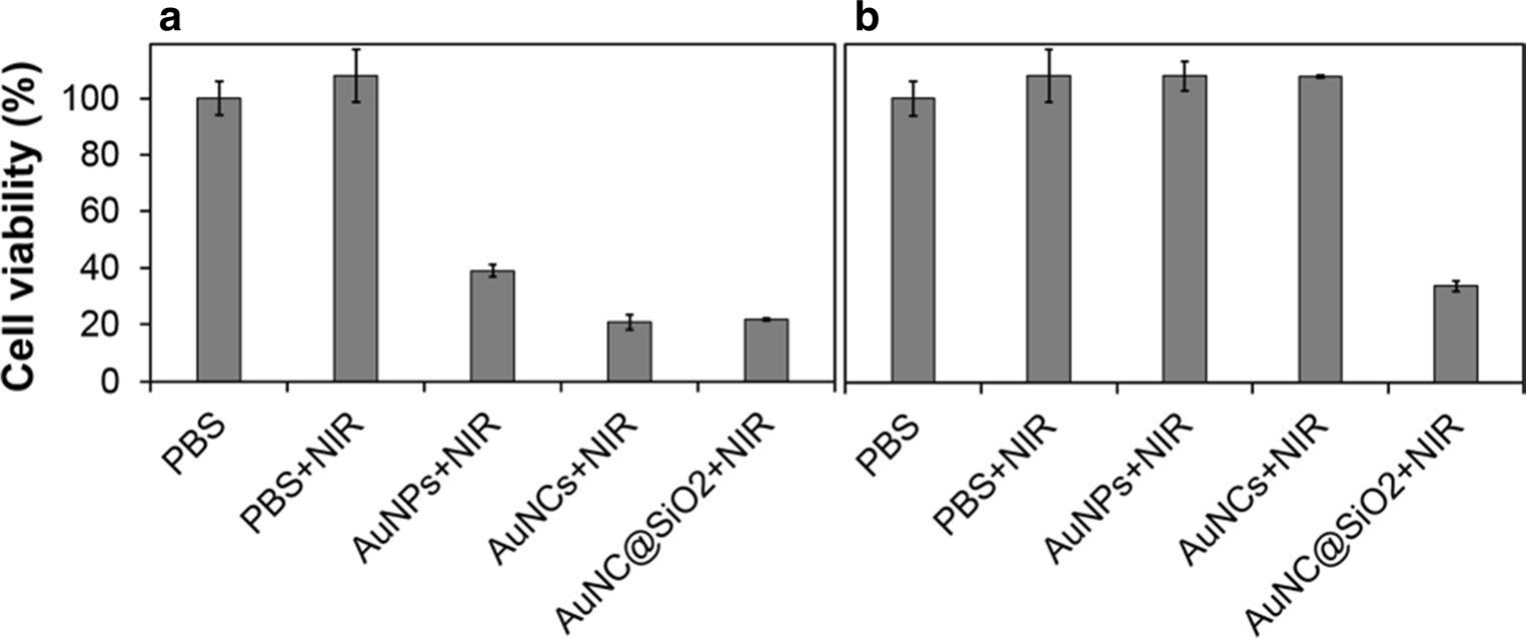
Photothermal efficiency of each sample in four different conditions. Histogram (**a**) shows cell viability when using the samples immediately after fabrication. Histogram (**b**) cells cultured in PBS for 7 days. Cells were subjected to 808 nm wavelength NIR irradiation after being cultured in PBS, AuNP, AuNC, and AuNC@SiO_2_. All measurements were done in triplicate

Application of AuNC@SiO_2_ as a photothermal agent in cancer treatment was demonstrated in vitro and even in vivo using the PC-3 cell line. The toxicity of the samples to the cell line was evaluated prior to this experiment. Additional file 1: Figure S7 showed that although the cellular viability upon exposure to various types and the amounts of AuNPs, AuNCs and AuNC@SiO_2_ was slightly different. All samples showed a survival rate of more than 70%. According to the International Organization for Standardization (ISO), a substance is defined as cytotoxic if the cell survival rate is less than 70% [46]. Therefore, it can be concluded that these gold nanomaterials did not show cytotoxicity to PC-3 cells after 24 h of exposure. Before irradiation during PTT of cancer cells, optimization of various parameters including incubation time, particle concentration, laser power, and irradiation time were given priority (Additional file 1: Figure S8). For this purpose, the death of cancer cells was observed as the combined effects of particles and the NIR laser energy, as shown in Fig. 4a. When AuNCs and AuNC@SiO_2_ were used as photothermal agents, they had similar efficacy against cancer cells, about 80%. When these particles were stored in PBS for 1 week, only AuNC@SiO_2_ had excellent stability. Other particles were not stable and aggregated or precipitated during storage, as is shown in Fig. 4b. These results suggested that the silica coating of AuNCs improved their stability in storage under PBS and preserved their performance in NIR PTT against cancer cells.

**Fig. 5:**
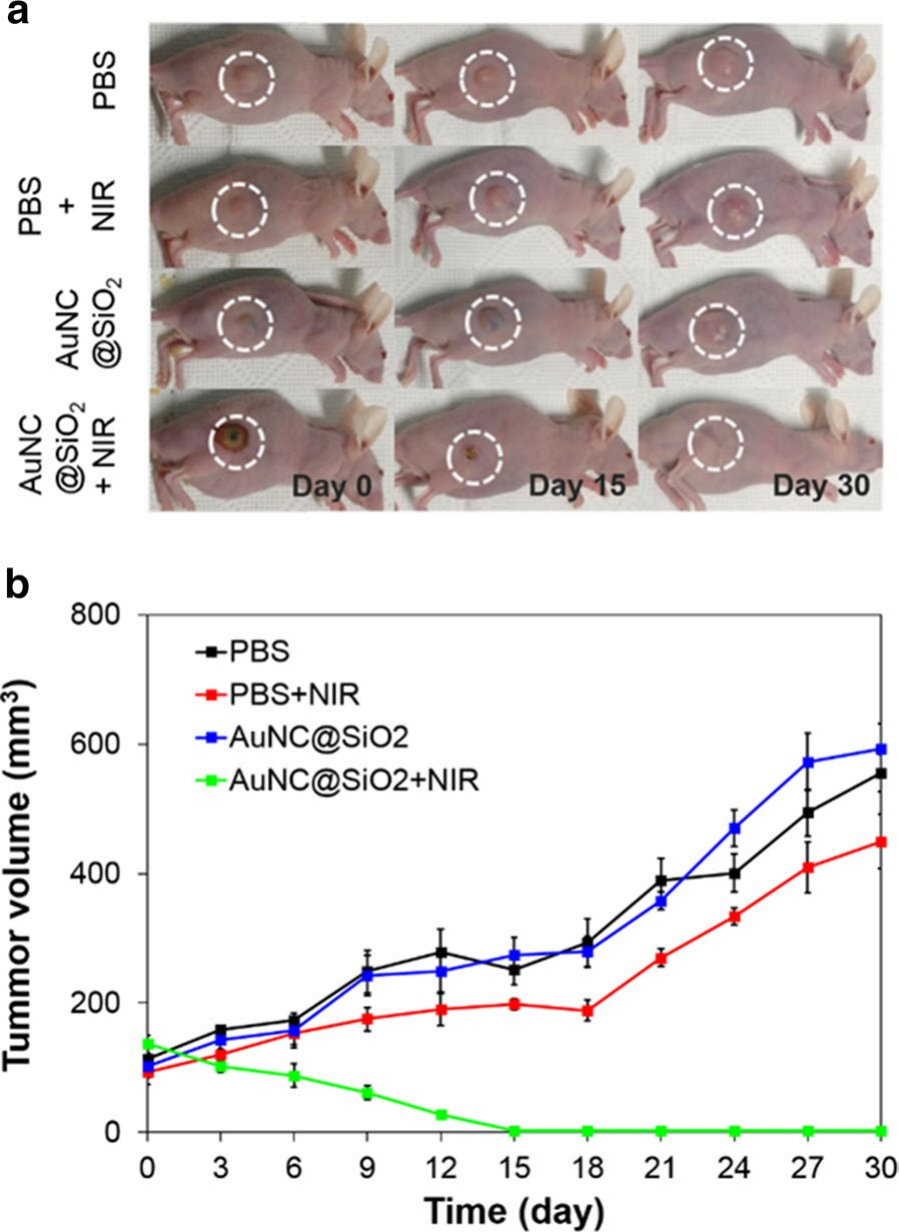
Images of the treated mice depended on time and trend of tumor growth. **a** Tumor size of AuNC@SiO_2_ PTT treated PC-3 tumor-implanted mice over time. Here, it is noted that the white dashed circle represents the tumor site. **b** Plot indicating the tumor size of AuNC@SiO_2_ PTT treated mice over time. All experimental measurements were made in triplicate

AuNC@SiO_2_ was finally tested on mice with tumors. The mice were reared for 3 weeks after PC-3 prostate cancer cells were injected into them and divided into four groups. In experimental group 1, the mice received neither laser irradiation nor particle injection as a control group. In group 2, they were subjected to laser irradiation and with no particle injections. The mice in group 3 were given particle injections but no laser irradiation. Mice in group 4 were exposed to both laser irradiation and particle injection. The tumor volume of all groups was measured every 3 days after treatment. Figure 5a shows an image of a mouse with a PC-3 xenograft 24 days after treatment. Group 4 mice showed black burn marks at the tumor site shortly after treatment. Alternatively, mice in other groups did not show any apparent changes at the tumor site after treatment. The marks in group 4 mice were caused by strong heat generated by the photothermal effect of AuNC@SiO_2_ under NIR irradiation. Over time, the marks gradually disappeared. The tumors became smaller until they, too, disappeared in group 4 mice. The sizes of the tumors observed in the other three groups are shown in Fig. 5b. Only group 4 mice, who received both laser irradiation and particle injections, showed a gradual decrease in tumor volume. It was clearly observed that the tumors in this group disappeared within 15 days. This strongly suggests that AuNC@SiO_2_ can serve as a potent NIR photothermal agent and effectively eliminate targeted prostate cancer cells.

## Conclusions

AuNC@SiO_2_ with a particle size diameter of 60–70 nm was synthesized, and its in vitro and in vivo applications as an NIR photothermal agent for prostate cancer treatment were tested. After the synthesis of gold nanoparticles and coating with a silica layer, the long-term stability of AuNCs was enhanced in PBS. FITC fluorescent dye was incorporated into the silica layer of AuNC@SiO_2_ and their cellular internalization by the PC-3 cell line was visualized and confirmed in target cells. The cytotoxicity of AuNC@SiO_2_ was also studied. It was not toxic to cells after 24 h, with similar results for AuNPs and uncoated AuNCs.

Next, an in vitro PTT test with PC-3 cancer cells was done using AuNC@SiO_2_ as a photothermal agent under 808-nm wavelength NIR. It demonstrated that they were effective in killing cancer cells. Lastly, AuNC@SiO_2_ were tested for in vivo cancer treatment. AuNC@SiO_2_ was intratumorally injected into tumor-bearing mice, which was followed by NIR irradiation at the tumor site. It was found that the mice with laser irradiation and injected AuNC@SiO_2_ showed black burn marks immediately after treatment due to strong heat generation from the photothermal effect of AuNC@SiO_2_ under NIR irradiation. Over time, tumor volume in these mice gradually decreased and the tumors eventually disappeared within 15 days. These results demonstrate the usefulness of AuNC@SiO_2_ as a photothermal agent in NIR cancer treatment. In the case of cancer near the skin, this treatment can be effective. Further studies may show the possibility of treating prostate cancer without surgery.

## Supplementary information

**Additional file 1: Figures S1 and S2.** The zeta potential and absorbance spectrum of each nanomaterial. **Figure S3.** More explanation about the calculation of photothermal transduction efficiency. **Figure S4.** The materials stability in PBS. **Figure S5.** The fabrication and characterization of FITC-AuNC@SiO_2_. **Figure S6.** The cellular internalization of FITC-AuNC@SiO_2_. **Figure S7.** The cytotoxicity test. **Figure S8.** The PTT test.

## Data Availability

Not applicable.
