# Supplementary material for: Gold nanoparticle clusters for the investigation of therapeutic efficiency against prostate cancer under near-infrared irradiation
Source: Nano Converg. 2020 Feb 17;7:5. doi: 10.1186/s40580-019-0216-z (PMC7024685; doi:10.1186/s40580-019-0216-z)
Supplement: Supplementary file 1 — Additional file 1: Figures S1 and S2. The zeta potential and absorbance spectrum of each nanomaterial. Figure S3. More explanation about the calculation of photothermal transduction efficiency. Figure S4. The materials stability in PBS. Figure S5. The fabrication and characterization of FITC-AuNC@SiO2. Figure S6. The cellular internalization of FITC-AuNC@SiO2. Figure S7. The cytotoxicity test. Figure S8. The PTT test. [file 40580_2019_216_MOESM1_ESM.doc]

Supporting Information for

**Gold Nanoparticle Clusters for the Investigation of Therapeutic Efficiency against Prostate Cancer under Near-Infrared Irradiation**

Jeonghun Kim, ^†^ Sang Hun Chun,^†^ Lunjakorn Amornkitbamrung, ^ζ^ Chanyoung Song, ^†, ‡^ Ji Soo Yuk, ^†^ So Yeon Ahn, ^†^ Byung Woo Kim, ^†^ Yong Taik Lim, ^†, ‡, $^ Byung-Keun Oh,^¶^ and Soong Ho Um^†, ‡, $*^

**Abstract:** Gold particles have been widely used in the treatment of prostate cancer due to their unique optical properties, such as their light-heat conversion in response to near-infrared radiation. Due to well‑defined synthesis mechanisms and simple manufacturing methods, gold particles have been fabricated in various sizes and shapes. However, the low photothermal transduction efficiency in their present form is a major obstacle to practical and therapeutic uses of these particles. In the current work, we present a silica-coated gold nanoparticle cluster to address the therapeutic limit of single gold nanoparticles (AuNPs) and use its photothermal effect for treatment against PC-3, a typical prostate cancer. Due to its specific nanostructure, this gold nanocluster showed three times higher photothermal transduction efficiency than free single AuNPs. Moreover, while free single particles easily clump and lose optical properties, this silica-coated cluster form remained stable for a longer time in a given medium. In photothermal tests under near-infrared radiation, the excellent therapeutic efficacy of gold nanoclusters, referred to as AuNC@SiO_2_, was observed in a preclinical sample. Only the samples with both injected nanoclusters followed by photothermal treatment showed completely degraded tumors after 15 days. Due to the unique intrinsic biocompatibility and higher therapeutic effect of these silica-coated gold nanoclusters, they may contribute to enhancement of therapeutic efficacy against prostate cancer.

[*†*] J. Kim, S. H. Chun, J. S. Yuk, S. Y. Ahn, Prof. B. W. Kim, Prof. Y. T. Lim, Prof. S. H. Um
School of Chemical Engineering
Sungkyunkwan University
2066 Seobu-ro, Suwon, Gyeonggi-do 16419 (South Korea)
E-mail: [sh.um@skku.edu](mailto:sh.um@skku.edu)

[*ζ*] Dr. L. Amornkitbamrung

Polymer Engineering Laboratory, Department of Chemical Engineering,

Faculty of Engineering, Chulalongkorn University

254 Phayathai Road, Patumwan, Bangkok 10330 (Thailand)

[*‡*] Dr. C. Song, Prof. Y. T. Lim, Prof. S. H. Um
SKKU Advanced Institute of Nanotechnology (SAINT)
Sungkyunkwan University
2066 Seobu-ro, Suwon, Gyeonggi-do 16419 (South Korea)

[$] Prof. Y. T. Lim, Prof. S. H. Um
Biomedical Institute for Convergence at SKKU (BICS)
Sungkyunkwan University
2066 Seobu-ro, Suwon, Gyeonggi-do 16419 (South Korea)

[¶] Prof. B-K. Oh

Department of Chemical and Biomolecular Engineering

Sogang University
35, Baekbeom-ro, Mapo-gu, Seoul 04107 (South Korea)

Summary of Contents

**Figures S1&2 regarding the zeta potential and absorbance spectrum of each nanomaterial**

**Figure S3 and more explanation about the calculation of photothermal transduction efficiency**

**Figure S4 regarding the materials stability in PBS**

**Figure S5 regarding the fabrication and characterization of FITC-AuNC@SiO_2_**

**Figure S6 regarding the cellular internalization of FITC-AuNC@SiO_2_**

**Figure S7 regarding the cytotoxicity test**

**Figure S8 regarding the PTT test**

**References**

Figure S1. The zeta-potential of each nanomaterial. Data obtained by zeta sizer and each value is an average of 15 times scanning. All of data were obtained triplicate and samples were synthesized at the same time.

**
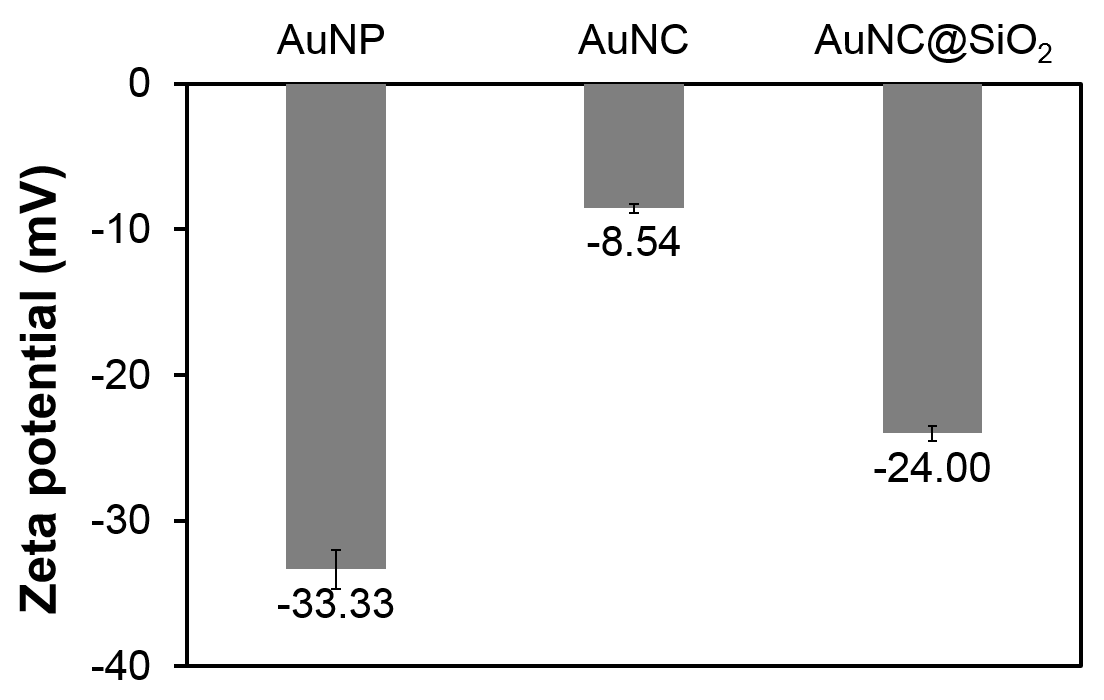
**

**Figure S2.** Absorbance spectrum of each material. This data was obtained by plate reader. All samples were synthesized at the same time.

**
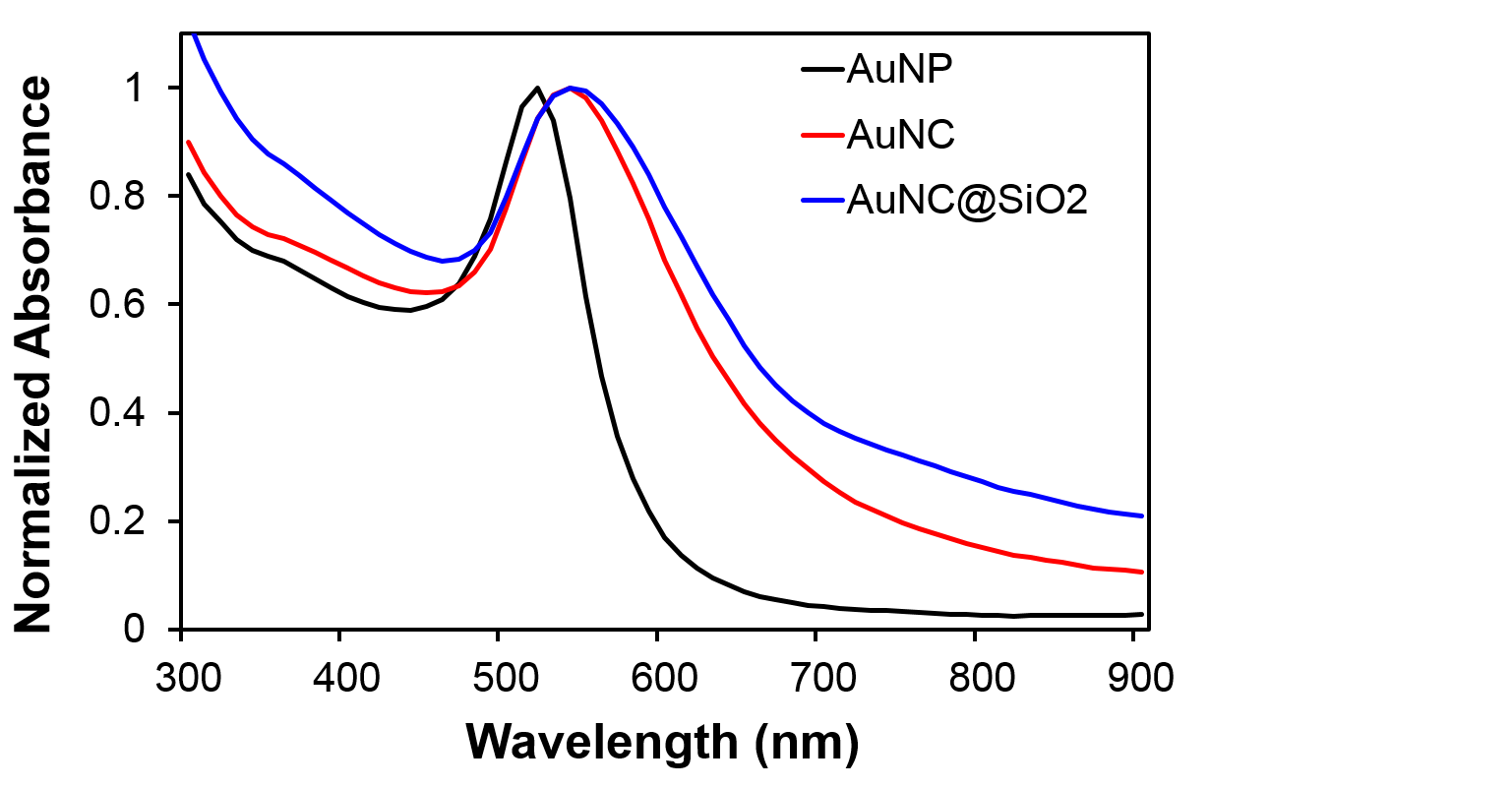
**

**Calculation of photothermal transduction efficiency (including Figure S3)**

The energy balance of the system is expressed by the following equation.^1-4^


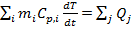
 (1)

where m_i_ is the mass of the system components, C_p,i_ is the heat capacity of the components and Q_j_ is the energy inside the system, where Q_NC_ is the energy dissipated by the nanomaterial, Q_Dis_ is the basic energy in the water, It is the heat energy that escapes out.


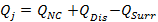
 (2)

Here, Q_NC_ can be expressed as follows.


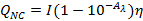
 (3)

*I* is the instantaneous output of the laser, A_λ_ is the absorption value at the wavelength band, and *η* is the thermal conversion efficiency of the material. Q_Surr_ can be expressed as the following expression.


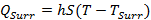
 (4)

Where *h* is the heat transfer coefficient and *S* is the surface area of the container. Here, the energy Q_Surr_ flowing out of the equilibrium state at T_Max_ in which the cell temperature does not rise any more is equal to Q_NC_ + Q_Dis_


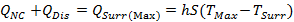
 (5)

Combined two equations (3) and (5), the photothermal transduction efficiency is defined as follows.


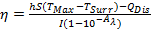
 (6)

To introduce the dimensionless driving force temperature and time, θ, τ_s_, to obtain the value of *hS*,


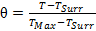
 (7)


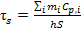
 (8)

When these two equations are applied to (1), we can summarize as follows.


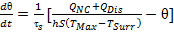
 (9)

Next, the relationship between θ and τ_s_ is defined as follows, and *hS* can be obtained from the following equation by measuring the temperature drop with time after the laser source is turned off.


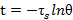
 (10)

Since the majority of the components in the container are water, so the value of
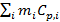
 is 8.4 J/℃ at m = 2 g, Cp = 4.2 J/g∙℃, and absorbance at 808 nm is 1.76 (
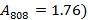
. Since I = 1W, *η* can be obtained by substituting each value.

**
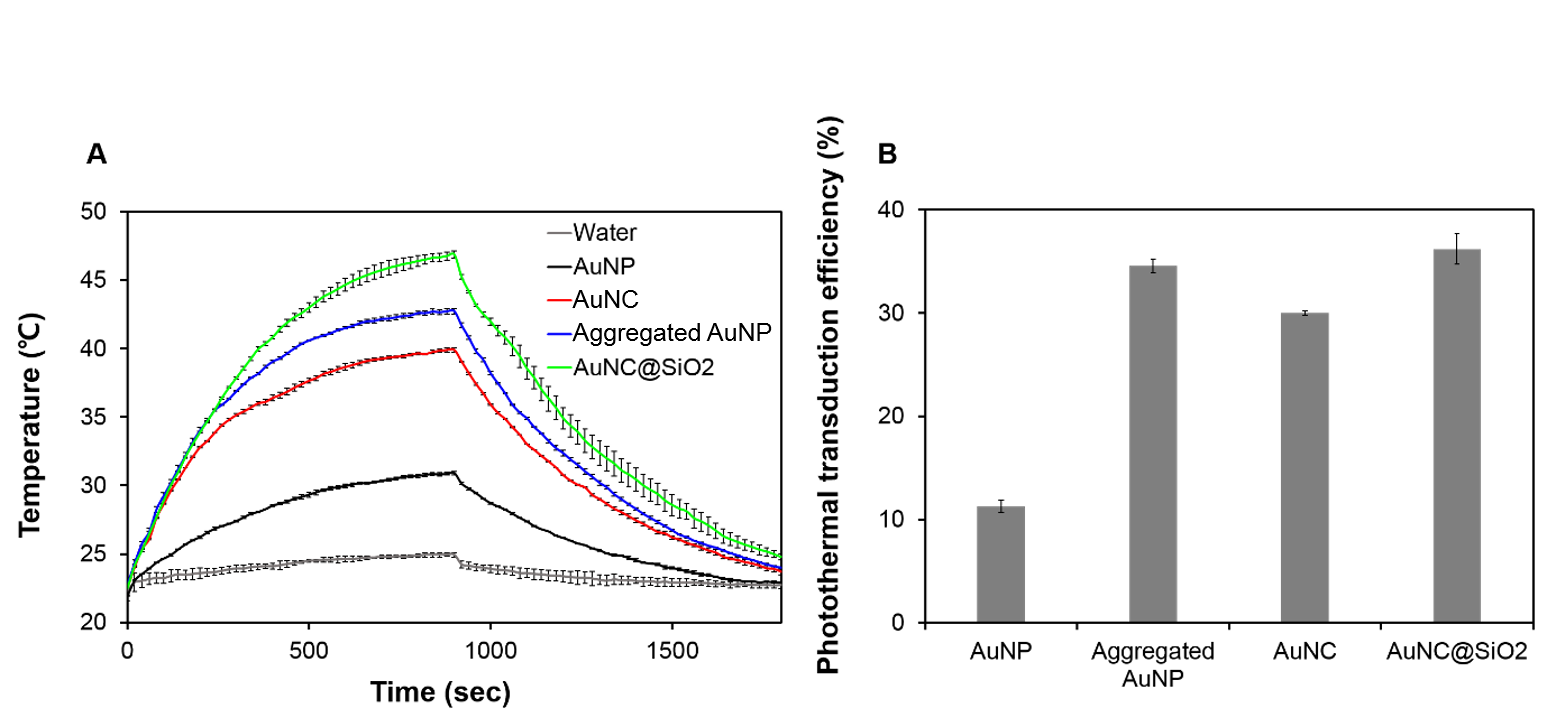
**

**Figure S3.** (A) is graph of temperature changes for each material when laser irradiation was applied and after irradiation was stopped. The power of the laser was fixed at 1 W and each experiment was carried out by triplication. (B) is calculated photothermal transduction efficiencies of each particle.

Figure S4. Change in absorption spectrum of each Au material in PBS at certain times; (A) AuNP, (B) AuNC and (C) AuNC@SiO_2_.


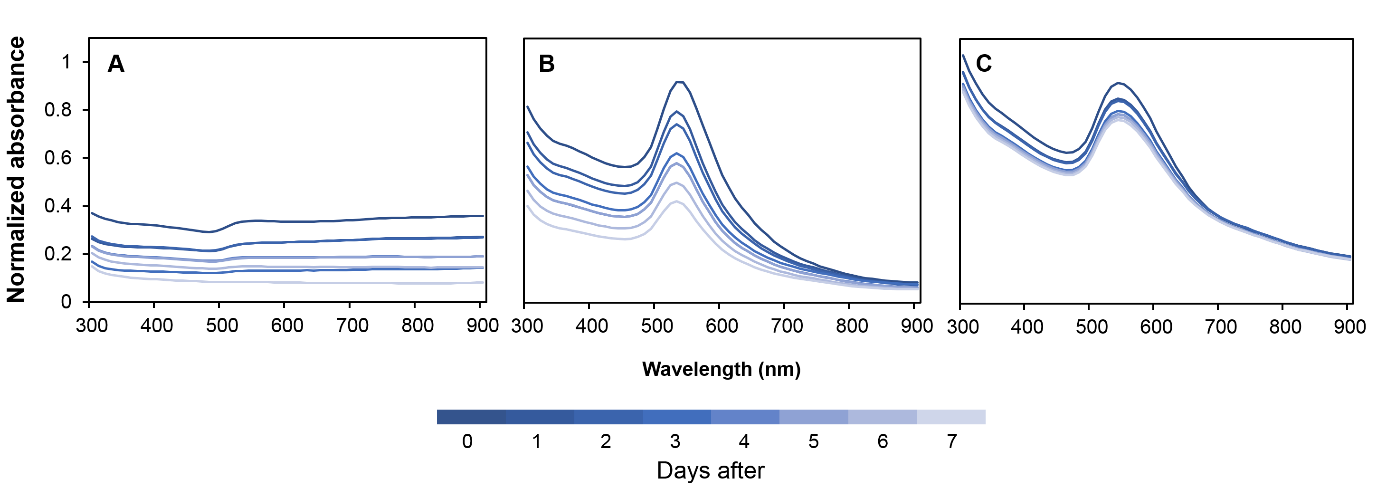


Figure S5. Synthesis and characterization of FITC-AuNC@SiO_2_. (A) is the synthesis process of FITC-AuNC@SiO_2_. (B) presents a comparison of absorption spectra of AuNC@SiO_2_, FITC-APTES and FITC-AuNC@SiO_2_. (C) shows the fluorescence intensity of FITC-APTES, supernatant after each washing step, ethanol (control), AuNC@SiO_2_ and FITC-AuNC@SiO_2_.


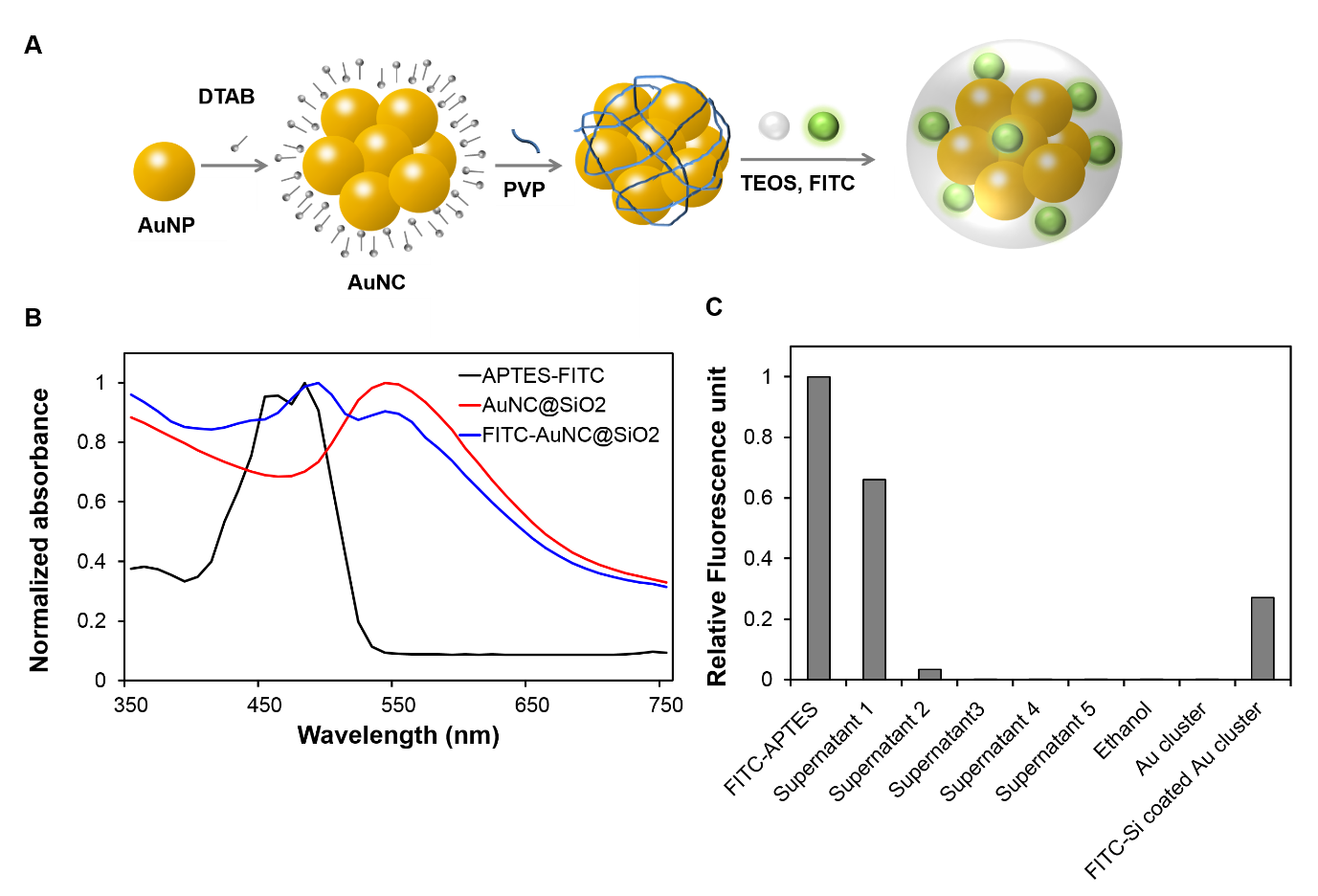


**Figure S6.** Mean fluorescence intensity data showing the cell infiltration of FITC-AuNC@SiO_2_ over time as measured by FACS. This graph shows the fluorescence of PC-3 cell treated with 20 μg/mL of AuNC@SiO_2_. Data obtained was done in triplicate.


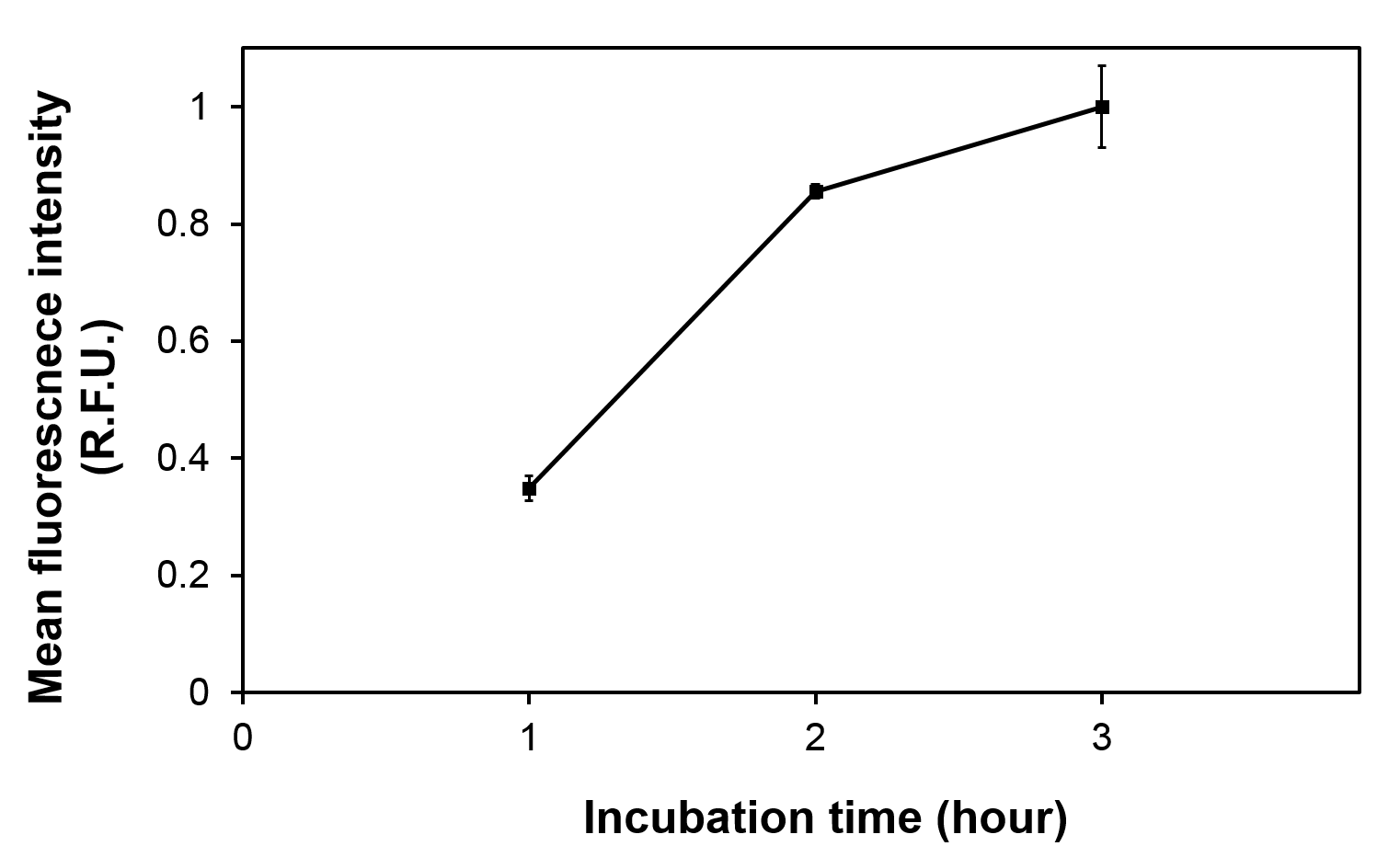


.

**Figure S7.** Cytotoxicity test with PC-3 cells. This graph shows the cellular toxicity results in various concentrations of (A) AuNP, (B) AuNC and the (C) AuNC@SiO_2_, for 24 hours against PC-3 cells. Data obtained was done in triplicate and unit is μg/mL.


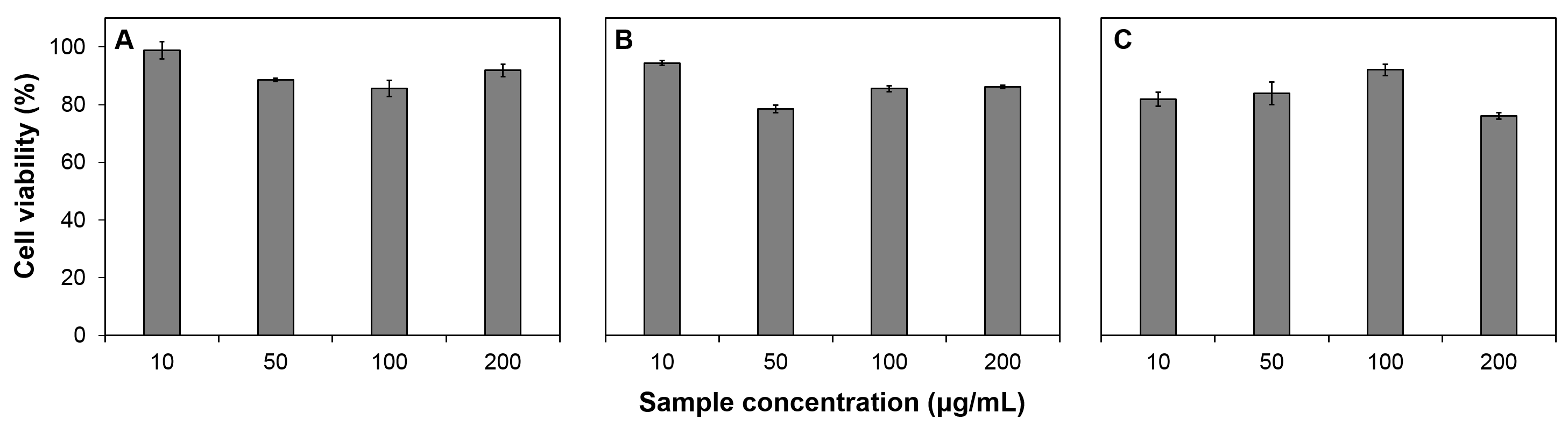


Figure S8. Preliminary results regarding parameter optimization of *in-vitro* photo-thermal treatments. (A) incubation time, (B) sample concentration, (C) laser power, and (D) irradiation time were changed under the base condition of 3 hours incubation time, 100 µg/mL particle concentration (silica coated gold nanoparticles clusters), 3 minutes irradiation, 2.5 W/cm^2^ laser power. All data obtained was done in triplicate.


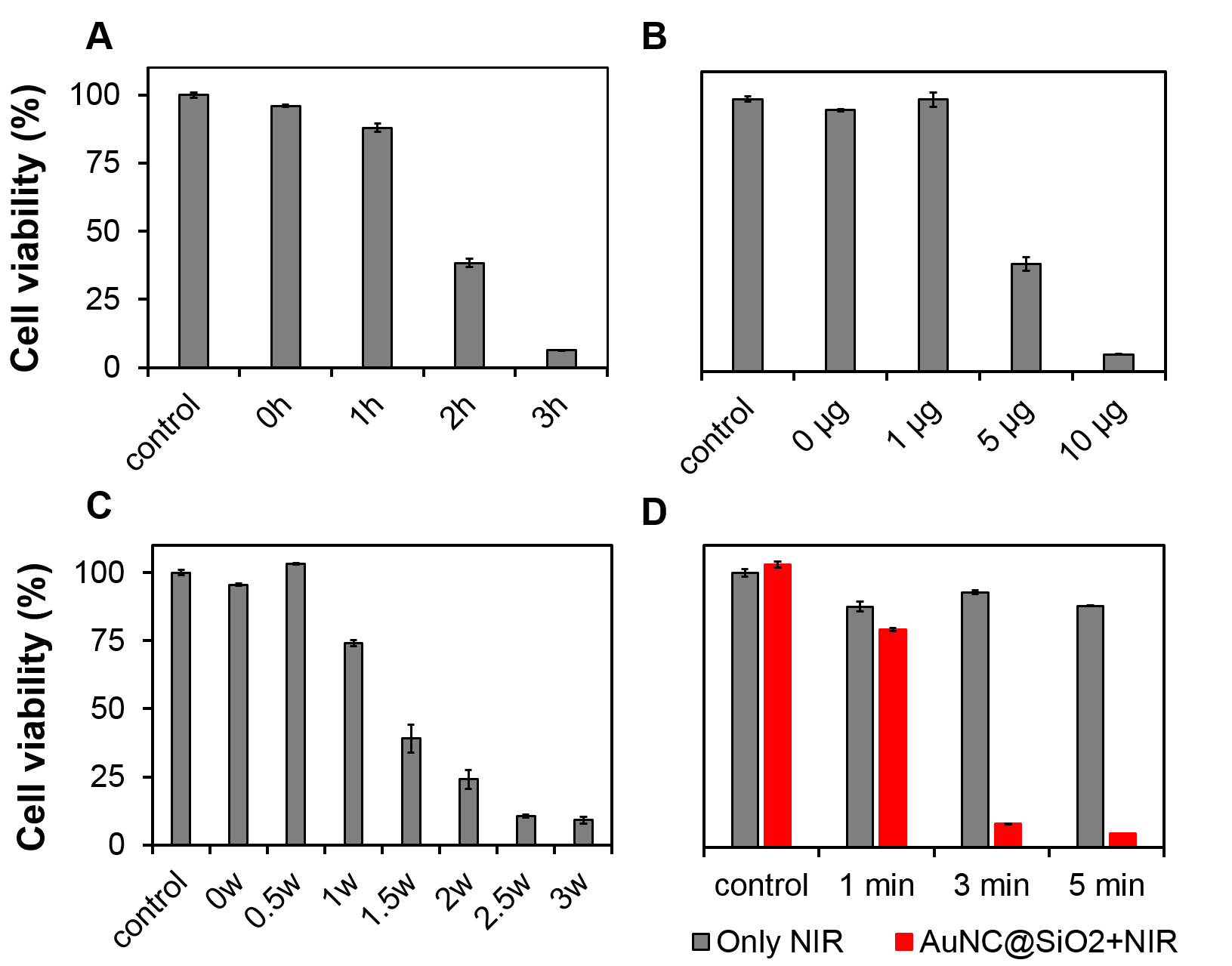


References

1. L. Amornkitbamrung, J. Kim, Y. Roh, S. H. Chun, J. S. Yuk, S. W. Shin, B.-W. Kim, B.-K. Oh, S. H. Um, Cationic Surfactant-Induced Formation of Uniform Gold Nanoparticle Clusters with High Efficiency of Photothermal Conversion under Near-Infrared Irradiation. Langmuir **34** (8), 2774-2783 (2018).
2. S. H. Liu, M .Y. Han, Synthesis, Functionalization, and Bioconjugation of Monodisperse, Silica-Coated Gold Nanoparticles: Robust Bioprobes. Adv. Funct. Mater. **15**(6), 961-967 (2005).
3. C. M. Hessel, V. P. Pattani, M. Rasch, M. G. Panthani, B. Koo, J. W. Tunnell, B. A. Korgel, Copper Selenide Nanocrystals for Photothermal Therapy. Nano Lett. **11**, 2560-2566 (2011).
4. Q. Tian, F. Jiang, R. Zou, Q. Liu, Z. Chen, M. Zhu, S. Yang, J. Wang, J. Wang, J. Hu, Hydrophilic Cu_9_S_5_ Nanocrystals: A Photothermal Agent with a 25.7% Heat Conversion Efficiency for Photothermal Ablation of Cancer Cells in Vivo. ACS Nano **5** (12), 9761-9771 (2011).
